# First Report on *Megaselia scalaris* Loew (Diptera: Phoridae) Infestation of the Invasive Pest *Spodoptera frugiperda* Smith (Lepidoptera: Noctuidae) in China

**DOI:** 10.3390/insects12010065

**Published:** 2021-01-13

**Authors:** Yunlin Tang, Qingyan Li, Li Xiang, Ruocheng Gu, Yanyan Wu, Yonghong Zhang, Xingrong Bai, Xiaohui Niu, Tian Li, Junhong Wei, Guoqing Pan, Zeyang Zhou

**Affiliations:** 1 State Key Laboratory of Silkworm Genome Biology, Southwest University, Chongqing 400715, China; t7anthony@email.swu.edu.cn (Y.T.); Li_qingyann@163.com (Q.L.); xiang163li@163.com (L.X.); guruocheng96@163.com (R.G.); wyy0711@email.swu.edu.cn (Y.W.); lit@swu.edu.cn (T.L.); zyzhou@swu.edu.cn (Z.Z.); 2 Chongqing Key Laboratory of Microsporidia Infection and Control, Southwest University, Chongqing 400715, China; 3 Key Laboratory of Sericultural Biology and Genetic Breeding, Ministry of Agriculture, Southwest University, Chongqing 400715, China; 4 School of Biotechnology, Southwest University, Chongqing 400715, China; 5 Institute of Sericulture and Apiculture, Yunnan Academy of Agriculture Sciences, Mengzi 661101, China; zhang200503@126.com (Y.Z.); bxrong3@163.com (X.B.); 6 Crop Seed Management Station of Chongqing, Chongqing 401121, China; xiaohui749@126.com; 7 College of Life Sciences, Chongqing Normal University, Chongqing 401331, China

**Keywords:** *Spodoptera frugiperda*, invasive pest, natural enemies, *Megaselia scalaris*, pest management

## Abstract

**Simple Summary:**

The invasive pest *Spodoptera frugiperda* first emerged in China in 2019, and therefore the information on indigenous natural enemies of *S. frugiperda* has been limited in China. In this study, we reported that a dipteran species was observed to infest *S. frugiperda* collected from maize fields in four different regions of China. Further morphological and molecular recognition identified all the flies as *Megaselia scalaris*. The findings of this study will improve our understanding on natural enemies to *S. frugiperda* and potentially provide new ideas for integrated pest management strategies in China.

**Abstract:**

The invasive pest *Spodoptera frugiperda* first emerged in China in January 2019 and has, to date, migrated to 29 provinces and municipalities in China, causing heavy crop damage in large areas. As a response to this invasive species from the environment, some indigenous natural enemies have been discovered and reported after *S. frugiperda* invasion. In this paper, parasitic flies were collected and identified from *S. frugiperda* collected in the Yunnan, Guangxi, and Henan provinces and the Chongqing municipality in China. By using both conventional and molecular approaches, we were able to show that all the parasitic flies of *S. frugiperda* identified in the four regions were *Megaselia. scalaris,* and that they attacked the pest larvae and pupae. This is the first report on an indigenous Chinese *Megaselia* species that has parasitic ability against the invasive pest *S. frugiperda*, potentially providing new ideas for pest control in China.

## 1. Introduction

As a major migratory agricultural pest, *Spodoptera frugiperda* originated in the Americas and migrated to the African continent in 2016. It was first discovered and reported in China in January 2019 [1,2]. With favorable temperatures and abundant crops, *S. frugiperda* rapidly spread to 29 provinces (autonomous regions, municipalities) across the country, posing a serious threat to food production security in China [3]. To control this new invasive and rapidly spreading pest, the “2019 *Spodoptera frugiperda* prevention and control technology plan (trial)” developed by Ministry of Agriculture and Rural Affairs of the People’s Republic of China recommended employing the chemical pesticides chlorantraniliprole, cyfluthrin, and deltamethrin for emergency control. In the following years, the pest may continue to cause seasonal outbreaks in China. The climate in southern China can support the presence *S. frugiperda* through the winter and this region could potentially be the annual breeding area for the pest [3]. In addition to emergency control by chemical pesticides, there is an urgent need for a long-term integrated pest management (IPM) strategy for *S. frugiperda* control. Biological control and natural enemy protection and utilization should be given priority, in addition to efforts towards reducing the number of applications and risks of pesticides [4].

Many kinds of potentially natural candidates for biological control, including entomophagous insects and entomopathogens, have been identified and reported throughout the world [5]. Investigations conducted in the Americas on the inventory of parasitoids and parasites of *S. frugiperda* have shown that approximately 150 species from 14 families are able to attack *S. frugiperda* [6,7]. Moreover, virus-based insecticides such as *S. frugiperda* multiple nucleopolyhedrovirus (*Sf*MNPV) and granulovirus (*Sf*GV) have been developed for *S. frugiperda* [8,9]. *S. frugiperda* has exhibited resistance to many commonly used entomopathogenic bacteria and fungi such as *Bacillus thuringiensis* and *Beauveria bassiana* [10,11]. The information on indigenous natural enemies of *S. frugiperda* has been limited in China because this species of pest only recently migrated to China (2019). Within the past year, several surveys on the natural enemies attacking *S. frugiperda* have been conducted in many regions of China, showing that a number of parasitic wasps and flies are able to attack *S. frugiperda*. These include *Telenomus remus*, *Diadegma semiclausum*, and *Exorista japonica* [12,13]. 

In this study, we serendipitously found that *S. frugiperda* was parasitized by some flies from four regions in China, and identified these flies as being of the species *Megaselia scalaris* by DNA barcoding [14,15]. This is the first report showing that *M. scalaris* could parasitize *S. frugiperda* in China. Our discovery expands the repertoire of indigenous natural enemies against *S. frugiperda,* and can ultimately contribute to the development of IPM strategies for pest control in China. 

## 2. Materials and Methods 

### 2.1. Parasitic Fly Collections 

**Figure 1:**
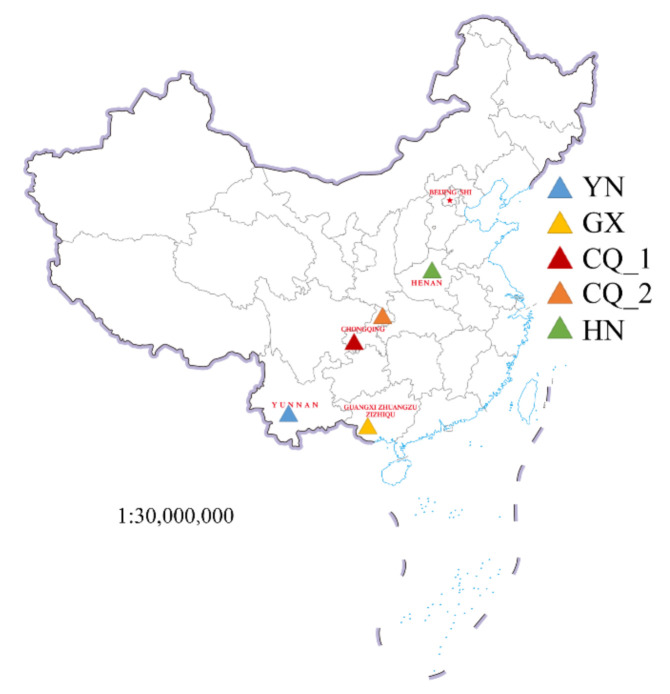
Sampling sites of parasitic flies and *Spodoptera. frugiperda* in China (Yunnan (YN), Guangxi (GX), Chongqing (CQ_1, CQ-2), and Henan (HN)).

In this study, five groups (YN, GX, CQ_2, HN, and CQ_1) of parasitic flies infesting *S. frugiperda* were collected from four regions in China (Figure 1). Of these, four were found in containers of *S. frugiperda* collected in Yunnan (YN), Guangxi (GX), Chongqing (CQ_2), and Henan (HN). The remaining group was found in the culture medium for *S. frugiperda* intestinal fungi isolation and was designated as CQ_1. Flies were captured with a combination of sweep netting over the container of *S. frugiperda* and maggots were collected into tubes directly. For each group 30–50 flies were collected, and 24 maggots were collected for the CQ_1 group. Following collection, flies and maggots were anesthetized over ice. 

### 2.2. Scanning Electron Microscopy (SEM)

The anesthetized flies were washed by sterilized phosphate buffer saline (PBS) and preserved in 75% ethanol for 24 h. They were then put in a critical point-drier and fixated with double-side adhesive tape, and then coated with gold in vacuum condition. Subsequently, electron microscopy observations were conducted to observe details with regard to the mouthpart, compound eyes, wing, and terminalia for morphological identification with a Hitachi SU3500 Scanning Electron Microscope. 

### 2.3. DNA Extraction and Gene Amplification 

The CTAB method was employed to extract genomic and mitochondrial DNA of parasitic flies [16]. Two pairs of DNA barcode primers were used to amplify the 28S rRNA gene (F: 5′-GACTACCCCCTGAATTTAAGCAT-3′ and R: 5′-GACTCCTTGGTCCGTGTTTCAAG-3′) and cytochrome oxidase subunit I (*CO*I) (F: 5′-GGTCAACAAATCATAAAGATATTGG-3′ and R: 5′-TAAACTTCAGGGTGACCAAAAAATCA-3′) gene of flies [14,15]. The PCR program was as follows: 96 °C for 8 min; 35 cycles at 96 °C for 40 s; 56 °C for 30 s; 72 °C for 1 min; and then 72 °C for 10 min. The specific PCR products were then assessed by 1.5% agarose gel electrophoresis and subsequently sequenced by Sangon Biotech (Shanghai, China) Co., Ltd. All sequences of the targeted genes in this study were submitted to NCBI under the accession number MT254753-MT254757 (28S rRNA), MT251290-MT251294 (*CO*I).

### 2.4. Data Analyses

The sequences of the 28S rRNA and COI gene of *S. frugiperda* were used to blast in the NCBI database. The top 100 sequences of each gene were downloaded and redundant low-quality sequences were removed after being aligned by MUSCLE v3.8.31 [17]. Phylogenetic trees were constructed using the neighbor-joining (NJ) method in MEGA5 [18]. 

## 3. Results

**Figure 2:**
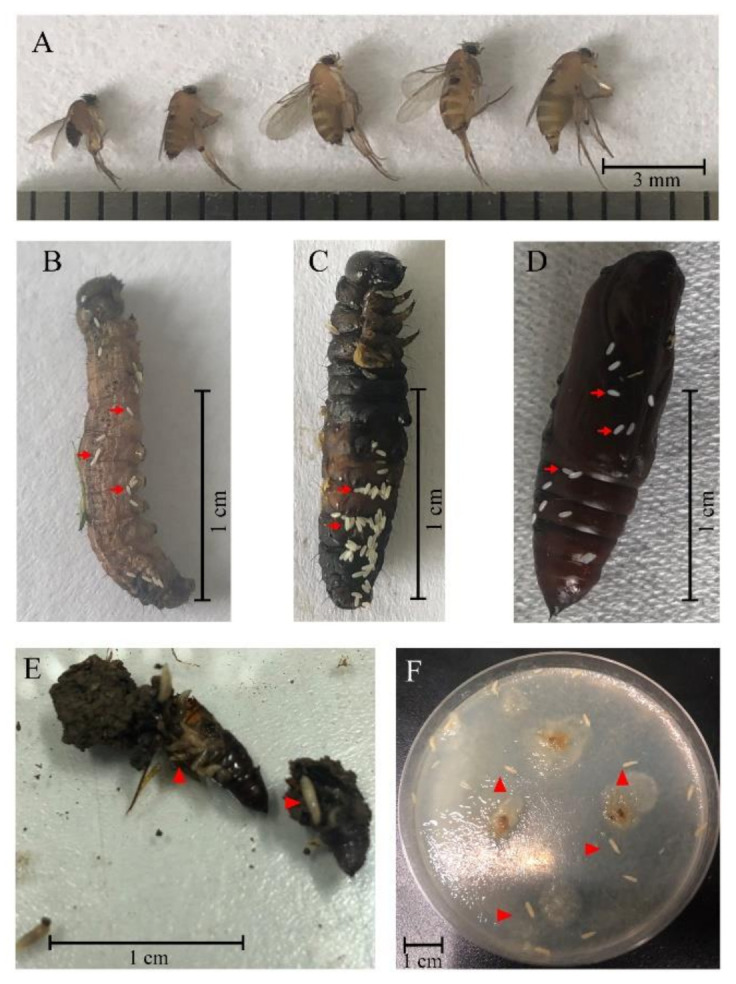
*Megaselia scalaris* specimens caught in *S. frugiperda* containers (**A**), *S. frugiperda* specimens with several eggs of *M.* *scalaris* (**B**–**D**), *M. scalaris* larvae emerging from the *S. frugiperda* pupa (**E**), and the medium for *S. frugiperda* intestinal fungi isolation (**F**). (**A**–**E**) were from Yunnan, and (**F**) originated in Chongqing. Red arrows indicate the eggs of *M.* *scalaris*, red triangles indicate the *M.* *scalaris* larvae.

**Figure 3:**
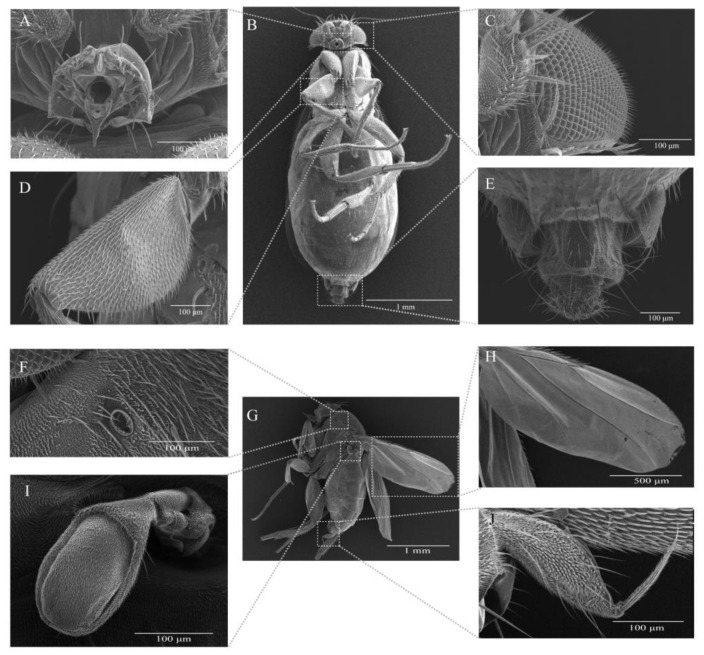
Scanning electron micrograph of the parasitic flies *M. scalaris* from Chongqing (female: (**A**–**E**), male: (**F**–**J**)). Female: (**A**) mouthpart, (**B**) segmental venter of female, (**C**) compound eyes, (**D**) tibia, and (**E**) female terminalia. Male: (**F**) spiracle, (**G**) left face of male, (**H**) wing, (**I**) haltere, and (**J**) male terminalia.

**Figure 4:**
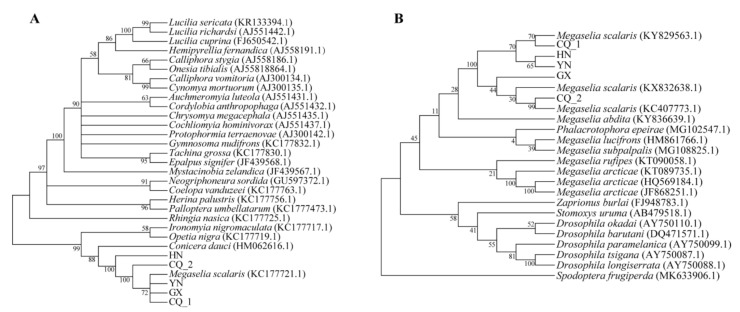
Phylogenetic tree of two DNA barcode sequences ((**A**) 28S rRNA, (**B**) *CO*I) of five *M. scalaris* specimens and comparative species.

In our surveillance studies, some flies (2.46 ± 0.50 by 0.75 ± 0.17 mm) were observed and caught in the closed but ventilated containers for *S. frugiperda* collected from the maize fields of four different regions in China (Figure 2A), and white long oval eggs (0.60 ± 0.04 by 0.2 ± 0.01 mm) were found on the epidermis of *S. frugiperda* larvae (Figure 2B,C) and pupae (Figure 2D). Moreover, we observed that the maggots emerged from thoracic cavities of *S. frugiperda* pupae, which were alive upon being collected from the maize fields, and left solid exoskeletons of *S. frugiperda* pupae as remnants (Figure 2E). Additionally, the maggots that emerged on the solid fungi culture medium (Potato Dextrose Agar Medium, PDA) were inoculated with the intestinal tract portion of *S. frugiperda* larvae (Figure 2F). To better characterize these flies morphologically, we next conducted a scanning electron microscopy (SEM) investigation (Figure 3), and concluded that these flies were of the species *M. scalaris* based on the presence of male terminalia from the SEM results (Figure 3J) [19]. To further confirm the identification of these flies by molecular approach, two pairs of universal DNA barcode primers were used to amplify the target genes from nuclear DNA and mitochondrial DNA of flies. The sequence alignments showed that all the parasite flies caught in different regions shared high homology in two DNA barcoding genes. The phylogenetic trees (Figure 4) constructed based on the 28S rRNA (Figure 4A) and *CO*I (Figure 4B) gene sequence show that the five fly specimens from this study have a high homologous evolutionary relationship with the Phoridae family, and clustered with *M. scalaris*.

## 4. Discussion

Since *S. frugiperda* invaded China, Chinese researchers have conducted investigations to identify natural enemies that are able to parasitize *S. frugiperda*. Egg parasitoid wasps [20,21] and some other natural enemies parasitizing *S. frugiperda* larvae were found and reported [12,13]. In this study, some dipterans infesting *S. frugiperda* were observed and caught in four regions of China. Morphological and DNA barcode identification confirmed that all the fly specimens collected in the four regions were of the species of *M. scalaris*.

*M. scalaris* is active in a wide range of geographical regions and usually feeds on decaying organic materials [22]. It has been reported in forensic cases [23], and there are some reports on the parasitism of *M. scalaris*. It has been reported that adult *M. scalaris* can break into the hive and lay eggs in the hive; the larvae of *M. scalaris* then hijack the food of bee larvae, therefore hindering the growth and development of bee larvae [24]. Koch and Costa found that laboratory-raised *Parastagmatoptera tessellata* and *Triatoma brasiliensis* could be parasitized by *M. scalaris* [25,26]; the larvae of *M. scalaris* can feed and grow in the host’s body. In our study, a similar phenomenon of *M. scalaris* parasitizing *S. frugiperda* in various regions of China was observed. The emergence of eggs on the epidermis of *S. frugiperda* larvae and pupae (Figure 2B–D), and maggots in the thoracic cavities of *S. frugiperda* pupae (Figure 2E) and the solid fungi culture medium inoculated with the intestinal tract portion of *S. frugiperda* larvae (Figure 2F) demonstrates that maggots can destroy the integrity of the epidermis and penetrate into the host body cavity to infest *S. frugiperda*. The emergence of flies (Figure 2A) in the closed but ventilated containers proves that the maggots are able to develop to the adult stage in the body cavity of *S. frugiperda*. All these results indicate that the indigenous species *M. scalaris* can parasitize the new invasive pest *S. frugiperda* in the wild in China. To our knowledge, this is the first record of *M. scalaris* infesting wild *S. frugiperda* in China. There have been a few previous reports of this fly infesting other insects in the wild [24,27,28,29]. Our finding indicates that *M. scalaris* may play various roles in the ecosystem in addition to being saprophages, such as being parasitic to insects.

In consideration of biological control to *S. frugiperda*, several native Chinese insects are reported to be parasitic and predatory to the new invasive pest *S. frugiperda*. However, their potential applications as biological control agents remain unclear and deserve further investigation. In terms of developing effective IPM strategies for controlling *S. frugiperda* in China, biological control, protection, and utilization of natural enemies should be given priority for reducing the use of pesticides. Therefore, there needs to be further investigation and exploitation of natural enemies in China to aid the development of subsequent IPM strategies.

## 5. Conclusions

In this study, we reported that some dipterans were able to infest invasive pest *S. frugiperda* in the wild in China. Both conventional and molecular methods identified the dipterans as *M. scalaris*. This is the first report on the native species *M. species* infesting *S. frugiperda* in China. Further investigations on the infestation of *S. frugiperda* by *M. scalaris* are warranted and such studies can potentially provide new directions for the integrated pest management strategies in China.

## Data Availability

All the data generated in this study have been submitted to NCBI for public availability under the accession number MT254753-MT254757 (28S rRNA), MT251290-MT251294 (*CO* I).
